# 2-[(1-Methyl-1*H*-pyrrol-2-yl)carbonyl­meth­yl]isoindoline-1,3-dione

**DOI:** 10.1107/S1600536809034515

**Published:** 2009-09-09

**Authors:** Joachim Schlosser, Dieter Schollmeyer, Stefan Laufer, Christian Peifer

**Affiliations:** a Institue of Pharmacy, Department of Pharmaceutical and Medicinal Chemistry, Eberhard-Karls-University Tuebingen, Auf der Morgenstelle 8, D-72076 Tuebingen, Germany; b Department of Organic Chemistry, Johannes Gutenberg University Mainz, Duesbergweg 10-14, D-55099 Mainz, Germany

## Abstract

The asymmetric unit of the title compound, C_15_H_12_N_2_O_3_, contains two almost identical mol­ecules forming an nearly *C*_2-_symmetric dimeric pattern. The dihedral angles between the pyrrole ring and the phthalimide unit are 82.95 (8) and 86.57 (8)° for the two mol­ecules. Within such a dimer, the phthalimide units of the two mol­ecules form a dihedral angle of 1.5 (5)°.

## Related literature

For regioselective synthesis of acyl­pyrroles see: Andersen & Exner (1977 ▶); Massa *et al.* (1990 ▶); Katritzky *et al.* (2003 ▶).
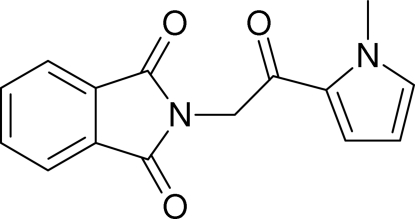


## Experimental

### 

#### Crystal data

C_15_H_12_N_2_O_3_
*M* *_r_* = 268.27
Monoclinic, 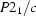

*a* = 10.8897 (7) Å
*b* = 14.8466 (4) Å
*c* = 15.8200 (9) Å
β = 101.619 (3)°
*V* = 2505.3 (2) Å^3^
*Z* = 8
Cu *K*α radiation
μ = 0.84 mm^−1^
*T* = 193 K
0.51 × 0.29 × 0.26 mm

#### Data collection

Enraf–Nonius CAD-4 diffractometer
Absorption correction: none
5001 measured reflections
4744 independent reflections
4400 reflections with *I* > 2σ(*I*)
*R*_int_ = 0.034
3 standard reflections frequency: 60 min intensity decay: 2%

#### Refinement

*R*[*F* ^2^ > 2σ(*F* ^2^)] = 0.045
*wR*(*F* ^2^) = 0.127
*S* = 1.05
4744 reflections
364 parameters
H-atom parameters constrained
Δρ_max_ = 0.34 e Å^−3^
Δρ_min_ = −0.27 e Å^−3^

### 

Data collection: *CAD-4 Software* (Enraf–Nonius, 1989 ▶); cell refinement: *CAD-4 Software*; data reduction: *CORINC* (Dräger & Gattow, 1971 ▶); program(s) used to solve structure: *SIR97* (Altomare *et al.*, 1999 ▶); program(s) used to refine structure: *SHELXL97* (Sheldrick, 2008 ▶); molecular graphics: *PLATON* (Spek, 2009 ▶); software used to prepare material for publication: *PLATON*.

## Supplementary Material

Crystal structure: contains datablocks I, global. DOI: 10.1107/S1600536809034515/bt5047sup1.cif

Structure factors: contains datablocks I. DOI: 10.1107/S1600536809034515/bt5047Isup2.hkl

Additional supplementary materials:  crystallographic information; 3D view; checkCIF report

## Figures and Tables

**Fig. 1.**
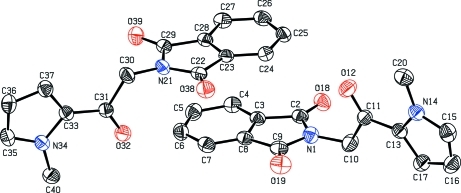
View of the title compound. Displacement ellipsoids are drawn at the 50% probability level. Hydrogen atoms omitted.

## supplementary crystallographic information

### Comment

Acylpyrroles are interesting building-blocks in the synthesis of therapeutic agents, *e.g.* in the preparation of small molecule drugs used for chemotherapy (Massa *et al.*, 1990). In line with this notion, regiospecific C-acylation of pyrroles in 2- or 3-position is a key for straightforward synthetic strategies of such organic molecules. As shown by Katritzky *et al.* (2003) regioselective 2-acylation of pyrroles can be achieved by using *N*-acylbenzotriazoles as auxiliar in the presence of TiCl_4_. Accordingly to this concept, the title compound was synthesized and we confirmed by crystal structure analysis the pyrrole system to be substituted in 2-position.

The symmetric crystal structure contains two highly similar molecules forming a dimeric pattern. The dihedral angle between the pyrrole and phtalimid moiety of molecule A is 82.95 (8)°, wheras molecule B is forming an angle of 86.57 (8)° between these aromatic systems. However, the N-phtalyl-moieties of molecule A and B, respectively, are forming a dihedral angle of 1.5 (5)°. The two crystallographic independend molecules form dimers by πi-πi-interactions. The distance between the centroid of the ring C3 - C8 (molecule A) and the least square plane C23 - C28 (molecule B) is 3.45 Å. A perspective view   of the title compound   is shown in Figure 1.

### Experimental

The title compound was synthesized following the general procedure for the preparation of 2-acylpyrroles established by Katritzky *et al.* (2003). Briefly, a solution of TiCl_4_ in CH_2_Cl_2 _(1.0 *M*, 17 ml) was added to a mixture of 1-methyl-1*H*-pyrrole (10,36 mmol) and 2-(2-(1*H*-benzo[*d*][1,2,3]triazol-1-yl)-2-oxoethyl) isoindoline -1,3-dione (8,3 mmol) in CH_2_Cl_2 _(20 ml) and stirred for 4 h at room temperature. The reaction was quenched by adding MeOH (5 ml). The solvents were evaporated under reduced pressure, and the residue was subjected to silica gel column chromatography using a hexane/ethylacetate gradient (80:20 - 70:30) as mobile phase to purify the product (yield: 67.5%). Crystals of the title compound were obtained by slow evaporation of a methanol solution at room temperature.

### Refinement

Hydrogen atoms   were placed at calculated positions with C—H = 0.95 Å (aromatic) or 0.98–0.99 Å (*sp*^3^ C-atoms). All H atoms were refined in the riding-model approximation with isotropic displacement parameters (set at 1.2–1.5 times of the *U*_eq_ of the parent atom).

### Crystal data

**Table:**

C_15_H_12_N_2_O_3_	*F*(000) = 1120
*M**_r_* = 268.27	*D*_x_ = 1.422 Mg m^−^^3^
Monoclinic, *P*2_1_/*c*	Cu *K*α radiation, λ = 1.54178 Å
Hall symbol: -P 2ybc	Cell parameters from 25 reflections
*a* = 10.8897 (7) Å	θ = 65–70°
*b* = 14.8466 (4) Å	µ = 0.84 mm^−^^1^
*c* = 15.8200 (9) Å	*T* = 193 K
β = 101.619 (3)°	Block, colourless
*V* = 2505.3 (2) Å^3^	0.51 × 0.29 × 0.26 mm
*Z* = 8	

### Data collection

**Table:**

Enraf–Nonius CAD-4 diffractometer	*R*_int_ = 0.034
Radiation source: rotating anode	θ_max_ = 69.9°, θ_min_ = 4.1°
graphite	*h* = 0→13
ω/2θ scans	*k* = 0→18
5001 measured reflections	*l* = −19→18
4744 independent reflections	3 standard reflections every 60 min
4400 reflections with *I* > 2σ(*I*)	intensity decay: 2%

### Refinement

**Table:**

Refinement on *F*^2^	Secondary atom site location: difference Fourier map
Least-squares matrix: full	Hydrogen site location: inferred from neighbouring sites
*R*[*F*^2^ > 2σ(*F*^2^)] = 0.045	H-atom parameters constrained
*wR*(*F*^2^) = 0.127	*w* = 1/[σ^2^(*F*_o_^2^) + (0.073*P*)^2^ + 1.0728*P*] where *P* = (*F*_o_^2^ + 2*F*_c_^2^)/3
*S* = 1.05	(Δ/σ)_max_ = 0.001
4744 reflections	Δρ_max_ = 0.34 e Å^−^^3^
364 parameters	Δρ_min_ = −0.27 e Å^−^^3^
0 restraints	Extinction correction: *SHELXL97* (Sheldrick, 2008), Fc^*^=kFc[1+0.001xFc^2^λ^3^/sin(2θ)]^-1/4^
Primary atom site location: structure-invariant direct methods	Extinction coefficient: 0.0026 (3)

### Special details

**Table:**

Geometry. All e.s.d.'s (except the e.s.d. in the dihedral angle between two l.s. planes) are estimated using the full covariance matrix. The cell e.s.d.'s are taken into account individually in the estimation of e.s.d.'s in distances, angles and torsion angles; correlations between e.s.d.'s in cell parameters are only used when they are defined by crystal symmetry. An approximate (isotropic) treatment of cell e.s.d.'s is used for estimating e.s.d.'s involving l.s. planes.
Refinement. Refinement of *F*^2^ against ALL reflections. The weighted *R*-factor *wR* and goodness of fit *S* are based on *F*^2^, conventional *R*-factors *R* are based on *F*, with *F* set to zero for negative *F*^2^. The threshold expression of *F*^2^ > σ(*F*^2^) is used only for calculating *R*-factors(gt) *etc*. and is not relevant to the choice of reflections for refinement. *R*-factors based on *F*^2^ are statistically about twice as large as those based on *F*, and *R*- factors based on ALL data will be even larger.

### Fractional atomic coordinates and isotropic or equivalent isotropic displacement parameters (Å^2^)

**Table:**

	*x*	*y*	*z*	*U*_iso_*/*U*_eq_	
N1	0.39270 (13)	0.34485 (10)	0.01591 (9)	0.0329 (3)	
C2	0.46171 (15)	0.26582 (11)	0.03592 (10)	0.0297 (3)	
C3	0.57346 (14)	0.29082 (10)	0.10221 (10)	0.0273 (3)	
C4	0.67252 (15)	0.23893 (11)	0.14345 (10)	0.0330 (4)	
H4	0.6763	0.1762	0.1323	0.040*	
C5	0.76675 (16)	0.28191 (12)	0.20197 (11)	0.0360 (4)	
H5	0.8368	0.2481	0.2309	0.043*	
C6	0.76016 (16)	0.37357 (12)	0.21878 (11)	0.0352 (4)	
H6	0.8259	0.4015	0.2588	0.042*	
C7	0.65856 (16)	0.42500 (11)	0.17785 (11)	0.0331 (4)	
H7	0.6531	0.4874	0.1901	0.040*	
C8	0.56604 (14)	0.38244 (10)	0.11897 (10)	0.0280 (3)	
C9	0.44834 (15)	0.41772 (11)	0.06494 (11)	0.0320 (4)	
C10	0.28288 (16)	0.35196 (13)	−0.05247 (11)	0.0383 (4)	
H10A	0.2792	0.2987	−0.0906	0.046*	
H10B	0.2911	0.4061	−0.0875	0.046*	
C11	0.16021 (15)	0.35816 (11)	−0.01983 (10)	0.0300 (3)	
O12	0.15704 (12)	0.33737 (9)	0.05414 (8)	0.0427 (3)	
C13	0.05263 (15)	0.38909 (10)	−0.08287 (10)	0.0294 (3)	
N14	−0.06493 (13)	0.40234 (9)	−0.06445 (9)	0.0320 (3)	
C15	−0.14245 (17)	0.43340 (12)	−0.13630 (12)	0.0397 (4)	
H15	−0.2284	0.4481	−0.1406	0.048*	
C16	−0.07732 (18)	0.44034 (13)	−0.20208 (12)	0.0427 (4)	
H16	−0.1098	0.4603	−0.2593	0.051*	
C17	0.04520 (17)	0.41260 (11)	−0.16894 (11)	0.0355 (4)	
H17	0.1117	0.4102	−0.1996	0.043*	
O18	0.43333 (11)	0.19337 (8)	0.00271 (8)	0.0375 (3)	
O19	0.40481 (12)	0.49318 (8)	0.06132 (9)	0.0428 (3)	
C20	−0.10471 (17)	0.38641 (12)	0.01728 (12)	0.0388 (4)	
H20A	−0.1907	0.4083	0.0129	0.058*	
H20B	−0.0486	0.4186	0.0637	0.058*	
H20C	−0.1015	0.3217	0.0299	0.058*	
N21	0.58245 (12)	0.42578 (9)	0.39592 (9)	0.0302 (3)	
C22	0.47025 (15)	0.43972 (10)	0.33749 (10)	0.0287 (3)	
C23	0.43484 (14)	0.35043 (10)	0.29679 (9)	0.0262 (3)	
C24	0.33207 (15)	0.32607 (11)	0.23521 (10)	0.0304 (3)	
H24	0.2701	0.3687	0.2106	0.036*	
C25	0.32325 (16)	0.23539 (11)	0.21062 (10)	0.0334 (4)	
H25	0.2539	0.2160	0.1680	0.040*	
C26	0.41323 (16)	0.17345 (11)	0.24692 (11)	0.0345 (4)	
H26	0.4044	0.1124	0.2288	0.041*	
C27	0.51668 (16)	0.19874 (11)	0.30963 (11)	0.0329 (4)	
H27	0.5785	0.1563	0.3349	0.040*	
C28	0.52509 (14)	0.28838 (10)	0.33324 (10)	0.0278 (3)	
C29	0.62031 (14)	0.33601 (11)	0.39818 (10)	0.0291 (3)	
C30	0.64309 (16)	0.49219 (11)	0.45774 (11)	0.0335 (4)	
H30A	0.6652	0.4638	0.5155	0.040*	
H30B	0.5833	0.5416	0.4612	0.040*	
C31	0.76166 (14)	0.53162 (10)	0.43435 (10)	0.0265 (3)	
O32	0.77978 (11)	0.52463 (8)	0.36106 (7)	0.0344 (3)	
C33	0.84253 (14)	0.57881 (10)	0.50392 (10)	0.0270 (3)	
N34	0.95340 (12)	0.62135 (9)	0.49670 (9)	0.0288 (3)	
C35	1.00335 (15)	0.66000 (11)	0.57314 (11)	0.0344 (4)	
H35	1.0793	0.6934	0.5852	0.041*	
C36	0.92692 (16)	0.64334 (12)	0.63070 (11)	0.0363 (4)	
H36	0.9402	0.6632	0.6889	0.044*	
C37	0.82680 (15)	0.59226 (11)	0.58807 (11)	0.0327 (4)	
H37	0.7594	0.5703	0.6121	0.039*	
O38	0.41466 (11)	0.51066 (8)	0.32588 (8)	0.0388 (3)	
O39	0.71219 (11)	0.30649 (9)	0.44629 (8)	0.0393 (3)	
C40	1.00841 (16)	0.62710 (12)	0.42003 (11)	0.0364 (4)	
H40A	0.9606	0.6701	0.3792	0.055*	
H40B	1.0060	0.5677	0.3928	0.055*	
H40C	1.0956	0.6474	0.4366	0.055*	

### Atomic displacement parameters (Å^2^)

**Table:**

	*U*^11^	*U*^22^	*U*^33^	*U*^12^	*U*^13^	*U*^23^
N1	0.0290 (7)	0.0358 (7)	0.0341 (7)	0.0020 (5)	0.0066 (5)	−0.0004 (6)
C2	0.0308 (8)	0.0310 (8)	0.0298 (8)	−0.0018 (6)	0.0123 (6)	−0.0003 (6)
C3	0.0284 (7)	0.0282 (7)	0.0272 (7)	−0.0024 (6)	0.0103 (6)	−0.0014 (6)
C4	0.0365 (9)	0.0283 (8)	0.0355 (8)	0.0024 (6)	0.0102 (7)	−0.0005 (6)
C5	0.0332 (8)	0.0402 (9)	0.0339 (8)	0.0037 (7)	0.0053 (7)	0.0016 (7)
C6	0.0329 (8)	0.0409 (9)	0.0316 (8)	−0.0050 (7)	0.0060 (6)	−0.0051 (7)
C7	0.0374 (9)	0.0281 (8)	0.0364 (8)	−0.0038 (6)	0.0132 (7)	−0.0052 (6)
C8	0.0293 (8)	0.0273 (8)	0.0303 (8)	−0.0007 (6)	0.0127 (6)	−0.0005 (6)
C9	0.0336 (8)	0.0307 (8)	0.0352 (8)	0.0018 (6)	0.0151 (7)	0.0014 (6)
C10	0.0325 (9)	0.0519 (10)	0.0304 (8)	0.0036 (7)	0.0056 (7)	0.0028 (7)
C11	0.0326 (8)	0.0277 (8)	0.0289 (8)	−0.0009 (6)	0.0047 (6)	−0.0002 (6)
O12	0.0387 (7)	0.0584 (8)	0.0311 (6)	0.0024 (6)	0.0069 (5)	0.0083 (6)
C13	0.0305 (8)	0.0254 (7)	0.0324 (8)	−0.0009 (6)	0.0064 (6)	−0.0012 (6)
N14	0.0308 (7)	0.0279 (7)	0.0374 (7)	−0.0002 (5)	0.0072 (6)	−0.0012 (5)
C15	0.0332 (9)	0.0356 (9)	0.0478 (10)	0.0039 (7)	0.0020 (7)	−0.0004 (7)
C16	0.0467 (10)	0.0408 (10)	0.0370 (9)	0.0044 (8)	0.0000 (8)	0.0048 (8)
C17	0.0393 (9)	0.0353 (9)	0.0316 (8)	0.0008 (7)	0.0068 (7)	0.0004 (7)
O18	0.0411 (7)	0.0339 (6)	0.0379 (6)	−0.0068 (5)	0.0089 (5)	−0.0080 (5)
O19	0.0436 (7)	0.0335 (6)	0.0538 (8)	0.0113 (5)	0.0158 (6)	0.0024 (5)
C20	0.0393 (9)	0.0369 (9)	0.0440 (10)	−0.0007 (7)	0.0171 (8)	−0.0028 (7)
N21	0.0304 (7)	0.0274 (7)	0.0325 (7)	−0.0070 (5)	0.0055 (5)	−0.0021 (5)
C22	0.0315 (8)	0.0258 (8)	0.0304 (8)	−0.0037 (6)	0.0100 (6)	0.0021 (6)
C23	0.0287 (7)	0.0257 (7)	0.0255 (7)	−0.0035 (6)	0.0087 (6)	0.0016 (6)
C24	0.0311 (8)	0.0320 (8)	0.0279 (8)	−0.0017 (6)	0.0061 (6)	0.0014 (6)
C25	0.0358 (8)	0.0353 (9)	0.0292 (8)	−0.0102 (7)	0.0072 (6)	−0.0037 (6)
C26	0.0440 (9)	0.0271 (8)	0.0351 (8)	−0.0071 (7)	0.0145 (7)	−0.0049 (6)
C27	0.0365 (9)	0.0274 (8)	0.0369 (8)	0.0020 (6)	0.0124 (7)	0.0005 (6)
C28	0.0273 (7)	0.0285 (8)	0.0292 (8)	−0.0026 (6)	0.0098 (6)	0.0011 (6)
C29	0.0266 (8)	0.0313 (8)	0.0303 (8)	−0.0031 (6)	0.0081 (6)	0.0019 (6)
C30	0.0348 (8)	0.0331 (8)	0.0333 (8)	−0.0100 (7)	0.0083 (7)	−0.0070 (7)
C31	0.0282 (7)	0.0200 (7)	0.0304 (8)	0.0014 (6)	0.0040 (6)	0.0004 (6)
O32	0.0391 (6)	0.0341 (6)	0.0305 (6)	−0.0056 (5)	0.0085 (5)	−0.0030 (5)
C33	0.0252 (7)	0.0232 (7)	0.0323 (8)	0.0004 (6)	0.0048 (6)	0.0003 (6)
N34	0.0264 (6)	0.0261 (6)	0.0338 (7)	−0.0007 (5)	0.0056 (5)	0.0001 (5)
C35	0.0287 (8)	0.0318 (8)	0.0399 (9)	−0.0027 (6)	0.0005 (7)	−0.0038 (7)
C36	0.0336 (8)	0.0414 (9)	0.0323 (8)	−0.0001 (7)	0.0028 (7)	−0.0079 (7)
C37	0.0289 (8)	0.0370 (9)	0.0322 (8)	−0.0010 (6)	0.0062 (6)	−0.0027 (7)
O38	0.0431 (7)	0.0255 (6)	0.0465 (7)	0.0015 (5)	0.0062 (5)	0.0008 (5)
O39	0.0294 (6)	0.0444 (7)	0.0415 (7)	0.0009 (5)	0.0014 (5)	0.0020 (5)
C40	0.0311 (8)	0.0417 (9)	0.0381 (9)	−0.0029 (7)	0.0108 (7)	0.0031 (7)

### Geometric parameters (Å, °)

**Table:**

N1—C2	1.395 (2)	N21—C22	1.391 (2)
N1—C9	1.396 (2)	N21—C29	1.393 (2)
N1—C10	1.446 (2)	N21—C30	1.451 (2)
C2—O18	1.210 (2)	C22—O38	1.210 (2)
C2—C3	1.485 (2)	C22—C23	1.490 (2)
C3—C4	1.378 (2)	C23—C24	1.376 (2)
C3—C8	1.391 (2)	C23—C28	1.385 (2)
C4—C5	1.391 (2)	C24—C25	1.399 (2)
C4—H4	0.9500	C24—H24	0.9500
C5—C6	1.391 (2)	C25—C26	1.382 (2)
C5—H5	0.9500	C25—H25	0.9500
C6—C7	1.392 (2)	C26—C27	1.394 (2)
C6—H6	0.9500	C26—H26	0.9500
C7—C8	1.380 (2)	C27—C28	1.380 (2)
C7—H7	0.9500	C27—H27	0.9500
C8—C9	1.485 (2)	C28—C29	1.484 (2)
C9—O19	1.213 (2)	C29—O39	1.2106 (19)
C10—C11	1.529 (2)	C30—C31	1.529 (2)
C10—H10A	0.9900	C30—H30A	0.9900
C10—H10B	0.9900	C30—H30B	0.9900
C11—O12	1.217 (2)	C31—O32	1.2191 (19)
C11—C13	1.452 (2)	C31—C33	1.445 (2)
C13—N14	1.383 (2)	C33—N34	1.388 (2)
C13—C17	1.392 (2)	C33—C37	1.390 (2)
N14—C15	1.353 (2)	N34—C35	1.350 (2)
N14—C20	1.463 (2)	N34—C40	1.460 (2)
C15—C16	1.376 (3)	C35—C36	1.374 (2)
C15—H15	0.9500	C35—H35	0.9500
C16—C17	1.394 (3)	C36—C37	1.387 (2)
C16—H16	0.9500	C36—H36	0.9500
C17—H17	0.9500	C37—H37	0.9500
C20—H20A	0.9800	C40—H40A	0.9800
C20—H20B	0.9800	C40—H40B	0.9800
C20—H20C	0.9800	C40—H40C	0.9800
			
C2—N1—C9	111.84 (13)	C22—N21—C29	112.03 (13)
C2—N1—C10	123.91 (14)	C22—N21—C30	124.51 (14)
C9—N1—C10	124.06 (15)	C29—N21—C30	122.72 (14)
O18—C2—N1	124.79 (15)	O38—C22—N21	125.16 (15)
O18—C2—C3	129.09 (15)	O38—C22—C23	129.10 (15)
N1—C2—C3	106.11 (13)	N21—C22—C23	105.72 (13)
C4—C3—C8	121.75 (15)	C24—C23—C28	121.84 (14)
C4—C3—C2	130.29 (15)	C24—C23—C22	129.99 (14)
C8—C3—C2	107.96 (13)	C28—C23—C22	108.16 (13)
C3—C4—C5	117.45 (15)	C23—C24—C25	116.77 (15)
C3—C4—H4	121.3	C23—C24—H24	121.6
C5—C4—H4	121.3	C25—C24—H24	121.6
C4—C5—C6	121.10 (16)	C26—C25—C24	121.44 (15)
C4—C5—H5	119.5	C26—C25—H25	119.3
C6—C5—H5	119.5	C24—C25—H25	119.3
C5—C6—C7	120.96 (15)	C25—C26—C27	121.34 (15)
C5—C6—H6	119.5	C25—C26—H26	119.3
C7—C6—H6	119.5	C27—C26—H26	119.3
C8—C7—C6	117.81 (15)	C28—C27—C26	116.89 (15)
C8—C7—H7	121.1	C28—C27—H27	121.6
C6—C7—H7	121.1	C26—C27—H27	121.6
C7—C8—C3	120.92 (15)	C27—C28—C23	121.71 (15)
C7—C8—C9	130.92 (15)	C27—C28—C29	130.24 (15)
C3—C8—C9	108.15 (14)	C23—C28—C29	108.03 (13)
O19—C9—N1	124.58 (16)	O39—C29—N21	124.42 (15)
O19—C9—C8	129.52 (16)	O39—C29—C28	129.53 (15)
N1—C9—C8	105.91 (13)	N21—C29—C28	106.03 (13)
N1—C10—C11	113.51 (14)	N21—C30—C31	112.88 (13)
N1—C10—H10A	108.9	N21—C30—H30A	109.0
C11—C10—H10A	108.9	C31—C30—H30A	109.0
N1—C10—H10B	108.9	N21—C30—H30B	109.0
C11—C10—H10B	108.9	C31—C30—H30B	109.0
H10A—C10—H10B	107.7	H30A—C30—H30B	107.8
O12—C11—C13	124.43 (15)	O32—C31—C33	125.11 (14)
O12—C11—C10	120.35 (14)	O32—C31—C30	120.25 (13)
C13—C11—C10	115.22 (14)	C33—C31—C30	114.58 (13)
N14—C13—C17	107.25 (14)	N34—C33—C37	106.88 (13)
N14—C13—C11	123.60 (14)	N34—C33—C31	124.40 (14)
C17—C13—C11	129.14 (15)	C37—C33—C31	128.71 (14)
C15—N14—C13	108.60 (14)	C35—N34—C33	108.67 (13)
C15—N14—C20	123.49 (15)	C35—N34—C40	124.11 (14)
C13—N14—C20	127.92 (14)	C33—N34—C40	127.20 (13)
N14—C15—C16	109.35 (16)	N34—C35—C36	109.18 (15)
N14—C15—H15	125.3	N34—C35—H35	125.4
C16—C15—H15	125.3	C36—C35—H35	125.4
C15—C16—C17	107.07 (16)	C35—C36—C37	107.34 (15)
C15—C16—H16	126.5	C35—C36—H36	126.3
C17—C16—H16	126.5	C37—C36—H36	126.3
C13—C17—C16	107.74 (16)	C36—C37—C33	107.93 (14)
C13—C17—H17	126.1	C36—C37—H37	126.0
C16—C17—H17	126.1	C33—C37—H37	126.0
N14—C20—H20A	109.5	N34—C40—H40A	109.5
N14—C20—H20B	109.5	N34—C40—H40B	109.5
H20A—C20—H20B	109.5	H40A—C40—H40B	109.5
N14—C20—H20C	109.5	N34—C40—H40C	109.5
H20A—C20—H20C	109.5	H40A—C40—H40C	109.5
H20B—C20—H20C	109.5	H40B—C40—H40C	109.5
			
C9—N1—C2—O18	179.77 (15)	C29—N21—C22—O38	176.76 (15)
C10—N1—C2—O18	−5.2 (2)	C30—N21—C22—O38	6.4 (2)
C9—N1—C2—C3	−1.54 (17)	C29—N21—C22—C23	−1.60 (17)
C10—N1—C2—C3	173.48 (14)	C30—N21—C22—C23	−171.96 (13)
O18—C2—C3—C4	−0.6 (3)	O38—C22—C23—C24	1.2 (3)
N1—C2—C3—C4	−179.19 (15)	N21—C22—C23—C24	179.45 (15)
O18—C2—C3—C8	178.89 (16)	O38—C22—C23—C28	−177.41 (16)
N1—C2—C3—C8	0.28 (16)	N21—C22—C23—C28	0.87 (16)
C8—C3—C4—C5	−1.0 (2)	C28—C23—C24—C25	−0.4 (2)
C2—C3—C4—C5	178.42 (15)	C22—C23—C24—C25	−178.77 (15)
C3—C4—C5—C6	0.7 (2)	C23—C24—C25—C26	0.3 (2)
C4—C5—C6—C7	0.4 (3)	C24—C25—C26—C27	0.0 (2)
C5—C6—C7—C8	−1.2 (2)	C25—C26—C27—C28	−0.3 (2)
C6—C7—C8—C3	1.0 (2)	C26—C27—C28—C23	0.3 (2)
C6—C7—C8—C9	−179.49 (15)	C26—C27—C28—C29	178.62 (15)
C4—C3—C8—C7	0.2 (2)	C24—C23—C28—C27	0.0 (2)
C2—C3—C8—C7	−179.38 (13)	C22—C23—C28—C27	178.75 (14)
C4—C3—C8—C9	−179.49 (14)	C24—C23—C28—C29	−178.60 (13)
C2—C3—C8—C9	0.98 (16)	C22—C23—C28—C29	0.11 (16)
C2—N1—C9—O19	−177.88 (15)	C22—N21—C29—O39	−176.96 (15)
C10—N1—C9—O19	7.1 (3)	C30—N21—C29—O39	−6.4 (2)
C2—N1—C9—C8	2.12 (17)	C22—N21—C29—C28	1.68 (17)
C10—N1—C9—C8	−172.89 (14)	C30—N21—C29—C28	172.23 (13)
C7—C8—C9—O19	−1.5 (3)	C27—C28—C29—O39	−1.0 (3)
C3—C8—C9—O19	178.12 (16)	C23—C28—C29—O39	177.49 (16)
C7—C8—C9—N1	178.52 (16)	C27—C28—C29—N21	−179.54 (16)
C3—C8—C9—N1	−1.88 (17)	C23—C28—C29—N21	−1.06 (16)
C2—N1—C10—C11	106.72 (18)	C22—N21—C30—C31	−107.89 (17)
C9—N1—C10—C11	−78.9 (2)	C29—N21—C30—C31	82.75 (18)
N1—C10—C11—O12	−16.2 (2)	N21—C30—C31—O32	18.2 (2)
N1—C10—C11—C13	164.30 (14)	N21—C30—C31—C33	−164.23 (13)
O12—C11—C13—N14	3.1 (3)	O32—C31—C33—N34	−1.2 (2)
C10—C11—C13—N14	−177.40 (15)	C30—C31—C33—N34	−178.61 (14)
O12—C11—C13—C17	−178.50 (17)	O32—C31—C33—C37	177.80 (16)
C10—C11—C13—C17	1.0 (3)	C30—C31—C33—C37	0.4 (2)
C17—C13—N14—C15	−0.31 (18)	C37—C33—N34—C35	−0.45 (17)
C11—C13—N14—C15	178.39 (15)	C31—C33—N34—C35	178.71 (14)
C17—C13—N14—C20	179.46 (15)	C37—C33—N34—C40	−178.90 (15)
C11—C13—N14—C20	−1.8 (3)	C31—C33—N34—C40	0.3 (2)
C13—N14—C15—C16	0.3 (2)	C33—N34—C35—C36	0.11 (19)
C20—N14—C15—C16	−179.47 (16)	C40—N34—C35—C36	178.62 (15)
N14—C15—C16—C17	−0.2 (2)	N34—C35—C36—C37	0.3 (2)
N14—C13—C17—C16	0.19 (19)	C35—C36—C37—C33	−0.6 (2)
C11—C13—C17—C16	−178.41 (16)	N34—C33—C37—C36	0.62 (18)
C15—C16—C17—C13	0.0 (2)	C31—C33—C37—C36	−178.50 (15)
